# Association of subclinical thyroid dysfunction with the risk of vertebral fracture: a meta-analysis of prospective cohort studies

**DOI:** 10.1080/07853890.2025.2558122

**Published:** 2025-09-11

**Authors:** Tao Zhang, Wenming Pan, Kai Huang, Jun Qiu, Wei Zhang, Jinhua Yang

**Affiliations:** Department of Spine Surgery, Changshu No.2 People’s Hospital, Changshu, Jiangsu, China

**Keywords:** Subclinical thyroid dysfunction, vertebral fracture, risk, systematic review, meta-analysis

## Abstract

**Background:**

Subclinical thyroid dysfunction (STD) is associated with an elevated risk of non-vertebral fractures. However, whether STD is associated with the risk of vertebral fracture remains controversial. This study aimed to determine the relationship between STD and the risk of vertebral fracture using a meta-analysis approach.

**Materials and methods:**

PubMed, Embase, and the Cochrane Library databases were searched for eligible studies published until March 01, 2025. Only prospective cohort studies that reported effect estimates with 95% confidence intervals (CIs) of vertebral fractures in participants with subclinical hyperthyroidism (SCH) and subclinical hypothyroidism (SH) compared to those with euthyroidism were included. A random-effects model was used to pool risk ratios (RRs), and analyses accounted for key covariates, including demographic factors, lifestyle variables, and disease history where reported.

**Results:**

Ten prospective cohort studies involving 61,219 individuals were included in this meta-analysis. SCH was associated with an increased risk of vertebral fracture (RR: 2.20; 95% CI: 1.60–3.02; *p* < 0.001). Moreover, the risk of vertebral fracture in individuals with SH was higher than that in those with euthyroidism (RR, 1.22; 95% CI: 1.01–1.49; *p* = 0.044). The pooled conclusions for the association between SCH and vertebral fracture risk were robust, whereas the significant association between SH and vertebral fracture was variable. The relationship between SH and vertebral fracture risk was affected by the median age of individuals (*p* = 0.047).

**Conclusion:**

Our study found that SCH was an independent risk factor for vertebral fracture, and that SH may increase the risk of vertebral fracture. Clinically, these findings support the need for regular monitoring of thyroid function, particularly in older adults, to identify individuals with STD who may benefit from targeted interventions to reduce vertebral fracture risk.

## KEY MESSAGES

We performed a systematic review and meta-analysis of prospective cohort studies to determine the relationship between subclinical thyroid dysfunction and risk of vertebral fractures.

Subclinical hyperthyroidism (SCH) and hypothyroidism (SH) are associated with an elevated risk of vertebral fractures.

The association between SCH and the risk of vertebral fracture was robust, whereas the median age of participants could affect the association between SH and the risk of vertebral fracture.

## Introduction

Osteoporosis is a widespread and concerning health problem globally. It is marked by low bone mass and a degraded micro-architecture of the bone tissue. This makes bones more brittle and greatly raises the risk of fractures [1]. Every year, around nine million fractures worldwide are caused by osteoporosis. This not only puts a heavy burden on healthcare systems but also increases the likelihood of morbidity and mortality, especially among women [2]. Vertebral fractures (VFs) are a prominent feature of osteoporosis. As the global population ages, the annual incidence of osteoporotic VFs is rising [3]. Previous studies has demonstrated that VF is an independent risk factor for future fractures [4]. Moreover, VFs can cause severe pain and spinal kyphosis, leading to disability and a significant decrease in the quality of life [5]. Identifying the risk factors for VFs is vital for reducing their occurrence.

Subclinical thyroid dysfunction (STD) refers to abnormal levels of thyroid-stimulating hormone (TSH), free triiodothyronine, and free thyroxine. Although these levels are still within the normal reference range, they indicate a potential thyroid issue. It has been reported that about 5% to 12% of adults have subclinical hyperthyroidism (SCH) and subclinical hypothyroidism (SH), respectively [6,7]. In nearly 60% of individuals, STD is a chronic condition, and it is more common in older adults [8–10]. Many studies have explored the connection between STD and bone-related outcomes. But the results have been inconsistent [11–15]. The main reason for this disparity is that STD involves subtle changes in thyroid function. In many studies, only a small number of participants experience thyroid dysfunction or fracture events. Several studies have examined the association between STD and the risk of fractures in different parts of the body. However, when it comes to VFs, the findings have been conflicting [16–20]. Some studies suggest a link between STD and VF risk, while others find no significant association. Some studies suggest a link between STD and VF risk, while others find no significant association.

Given the significant impact of osteoporosis and VFs on public health, understanding the related risk factors is crucial. STD, a common condition affecting a substantial portion of the adult population, has been studied in relation to bone health, but with inconsistent results. Thus, the current study aimed to use prospective cohort studies to accurately evaluate the association between STD and the specific risk of VF.

## Materials and methods

### Search strategy and selection criteria

This study was performed in accordance with the Preferred Reporting Items for Systematic Reviews and Meta-Analysis Statement [21], and it was registered on the INPLASY platform (no: INPLASY202350093). Prospective cohort studies investigating the association between STD and VF risk were eligible for this study, and no restrictions were placed on publication language or status. The PubMed, Embase, and the Cochrane Library databases were searched by us to identify potential studies for inclusion up to March 01, 2025. The medical subject headings of ‘thyroid, thyrotropin, hyperthyroidism, hypothyroidism, and fracture’ and keywords of ‘subclinical hypothyroidism, subclinical hyperthyroidism, subclinical thyroid dysfunction, and subclinical thyroid disorder’ were used. The details search strategy in PubMed is shown in File S1. The reference lists of the identified original and review articles were manually searched to identify any further studies that met the inclusion criteria. The literature search was mainly focused on article titles, study design, population, exposure, and outcomes.

The literature search and study selection processes were independently performed by two reviewers. Conflicting results were resolved through discussion with an additional reviewer until consensus was reached. A study was included if it met the following criteria: (1) participants: general population free of VF at baseline; (2) exposure: SCH or SH; (3) control: TSH within the normal range; (4) outcome: effect estimate (risk ratio [RR], hazard ratio, or odds ratio) with 95% confidence intervals (CIs) for comparisons of SCH or SH and euthyroidism on the risk of VF; and (5) study design: all studies had a prospective cohort study design. Retrospective observational studies were excluded because various confounding factors could have biased the results.

### Data collection and quality assessment

Two reviewers independently extracted the following information: first author and study group name, publication year, region, description of the study sample, sample size, proportion of males, number of cases of SCH and SH, thyroid medication users, follow-up duration, adjusted factors, and reported effect estimates. The methodological quality of the included studies was assessed using the Newcastle-Ottawa Scale (NOS), which has been partially validated for assessing the quality of observational studies in meta-analyses [22]. The NOS includes the domains of selection (representativeness of the exposed cohort, selection of the non-exposed cohort, ascertainment of STD, demonstration that outcomes was not present at start of study), comparability (comparability on the basis of the design or analysis), and outcome (assessment of outcome, adequate follow-up duration, and adequate follow-up rate), and the ‘star system’ of NOS ranges from to 0 to 9 for an individual study. Inconsistent results between the reviewers regarding data collection and quality assessment were adjudicated by an additional reviewer who examined the original article.

### Statistical analysis

The associations between STD and VF risk were examined based on the effect estimate and 95%CI in individual studies, and a random-effects model was applied to calculate the pooled RR and 95%CI, taking into account the underlying variation among the included studies [23,24]. The *I^2^* index and Cochran’s Q test were used to assess the heterogeneity across the included studies, and significant heterogeneity was defined as *I*^2^ > 50.0% or *p* < 0.10 [25,26]. The robustness of the pooled conclusion was assessed using sensitivity analysis through sequential removal of single studies [27]. Subgroup analyses of the association between STD and the risk of VF were performed according to sample size, median age, proportion of males, follow-up duration, adjusted level, and study quality, and the differences between subgroups were compared using a test of interaction [28]. The adjusted levels were categorized as high, moderate, and low. Factors adjusted at the high level included demographic variables, lifestyle variables, history of disease, thyroid-altering medication, and anti-osteoporotic medication. Factors adjusted at the moderate level included demographic variables, lifestyle variables, history of disease, and thyroid-altering medication. Factors adjusted at the low level included demographic variables and lifestyle variables. Publication bias was assessed using funnel plots, and Egger’s and Begg’s tests [29,30]. The trim-and-fill method was applied to adjust the conclusion if a significant publication bias was detected [31]. All reported *P*-values for pooled conclusions were two-sided, and *p* < 0.05 was regarded as statistically significant. All analyses were performed using the STATA software (version 12.0; StataCorp LLC, College Station, TX, USA).

## Results

### Literature search

**Figure 1.**
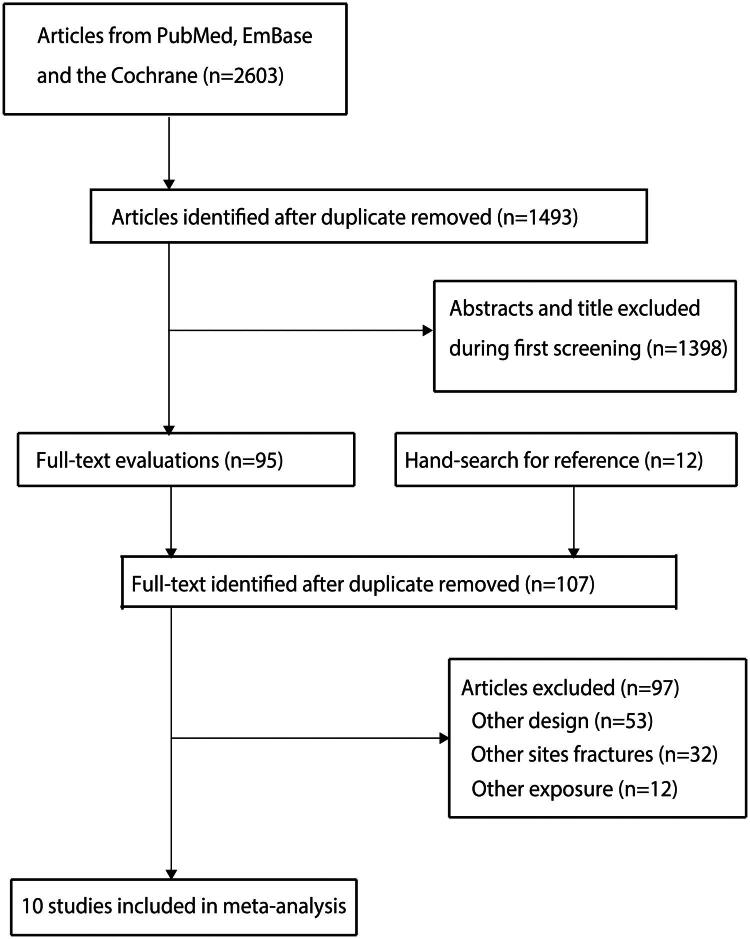
The PRISMA flowchart of the literature search and study selection process.

A total of 2,603 articles were initially identified, 1,493 of them were retained after excluding duplicate studies. Among these, 1,398 were excluded because their titles or abstracts were irrelevant. Reviewing the reference lists of the remaining 95 relevant articles led to the discovery of an additional 12 potential articles. Therefore, 107 articles were retrieved for full-text evaluation. Among these, 97 were excluded because they either did not have a prospective cohort design (*n* = 53), analyzed fractures at other sites (*n* = 32), or used different exposures (*n* = 12). The remaining ten prospective cohort studies were included in the meta-analysis [32–41]. The details of the study selection process are shown in Figure 1.

### Study characteristics

The general characteristics of the included studies and their participants are shown in Table S1. These studies included 61,219 individuals, with sample sizes ranging from 686 to 25,205. The median age of the included studies ranged from 51.0 to 75.4 years, and the follow-up duration ranged from 3.7 to 21.0 years. Two studies included only males, one included only females, and the remaining seven studies included both males and females. Four of the included studies were conducted in the USA, five in Europe, and one in Australia. The study quality was assessed using NOS: five studies received nine stars, three received eight stars, and two received seven stars (Table S1).

### Absolute risk of vertebral fracture across populations

During data extraction, we noted a key limitation: only one included study reported raw absolute VF incidence rates for SCH, SH, and euthyroid populations. All other included studies solely provided RR estimates for VF comparisons between STD (SCH/SH) and euthyroid groups, without disclosing absolute VF counts, person-time at risk, or incidence rates. Due to the absence of consistent absolute risk data across the remaining nine studies, we were unable to pool or aggregate absolute VF risk statistics for all three populations (euthyroid, SCH, SH) across the entire meta-analysis. This limitation should be considered when interpreting the relative risk results below.

### Subclinical hyperthyroidism and vertebral fracture risk

**Figure 2.**
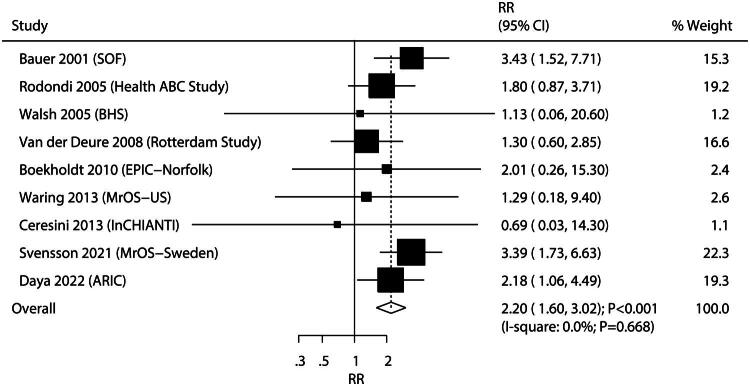
Association of subclinical hyperthyroidism with the risk of vertebral fracture.

Nine studies investigated the association between SCH and the risk of VF. The summary results indicated that SCH was associated with an increased risk of VF compared to euthyroidism (RR: 2.20; 95%CI: 1.60–3.02; *p* < 0.001; Figure 2), and no evidence of heterogeneity was observed across the included studies (*I^2^*=0.0%; *p* = 0.668). Sensitivity analysis indicated that the pooled conclusion was robust and not altered by the removal of any specific study (Figure S1). Subgroup analyses revealed that SCH was associated with an increased risk of VF in most subgroups, except in those with moderately adjusted levels or moderate quality (Table 1). Furthermore, the association between SCH and the risk of VF was not affected by sample size (*p* = 0.951), median age (*p* = 0.209), proportion of males (*p* = 0.293), follow-up duration (*p* = 0.527), adjusted level (*p* = 0.674), or study quality (*p* = 0.400).

**Table 1.** Subgroup analyses for the associations of SCH or SH with the risk of vertebral fracture.

Outcomes	Factors	Subgroups	Number of studies	RR and 95%CI	*P* value	I^2^ (%)	Q statistic	Interaction *P* value
SCH	Sample size	≥10,000	2	2.16 (1.09–4.27)	0.026	0.0	0.941	0.951
		<10,000	7	2.21 (1.55–3.17)	<0.001	0.0	0.445	
	Median age (years)	≥70.0	5	2.60 (1.73–3.91)	<0.001	0.0	0.520	0.209
		<70.0	4	1.71 (1.03–2.84)	0.037	0.0	0.798	
	Male (%)	≥50.0	3	2.93 (1.57–5.46)	0.001	0.0	0.535	0.293
		<50.0	6	1.99 (1.38–2.88)	<0.001	0.0	0.629	
	Follow-up duration (years)	≥10.0	4	1.95 (1.20–3.18)	0.007	0.0	0.965	0.527
		<10.0	5	2.33 (1.40–3.87)	0.001	22.2	0.273	
	Adjusted level	High	6	2.31 (1.57–3.40)	<0.001	0.0	0.804	0.674
		Moderate	3	1.95 (0.87–4.38)	0.106	39.9	0.189	
	Study quality	High	7	2.25 (1.63–3.10)	<0.001	0.0	0.536	0.400
		Moderate	2	0.89 (0.11–7.46)	0.918	0.0	0.820	
SH	Sample size	≥10,000	3	1.30 (1.09–1.54)	0.003	0.0	0.585	0.125
		<10,000	5	0.90 (0.58–1.40)	0.649	1.5	0.398	
	Median age (years)	≥70.0	3	0.77 (0.41–1.44)	0.411	15.0	0.309	0.047
		<70.0	5	1.30 (1.10–1.53)	0.002	0.0	0.880	
	Male (%)	≥50.0	2	0.92 (0.37–2.32)	0.866	0.0	0.985	0.531
		<50.0	6	1.23 (0.93–1.64)	0.151	29.5	0.214	
	Follow-up duration (years)	≥10.0	5	1.18 (0.84–1.64)	0.336	37.8	0.169	0.900
		<10.0	3	1.28 (0.70–2.33)	0.417	0.0	0.597	
	Adjusted level	High	5	1.06 (0.63–1.78)	0.818	38.1	0.167	0.564
		Moderate	3	1.26 (1.06–1.51)	0.010	0.0	0.711	
	Study quality	High	6	1.20 (0.92–1.55)	0.179	26.0	0.239	0.702
		Moderate	2	1.64 (0.38–7.08)	0.508	0.0	0.446	

### Subclinical hypothyroidism and vertebral fracture risk

**Figure 3.**
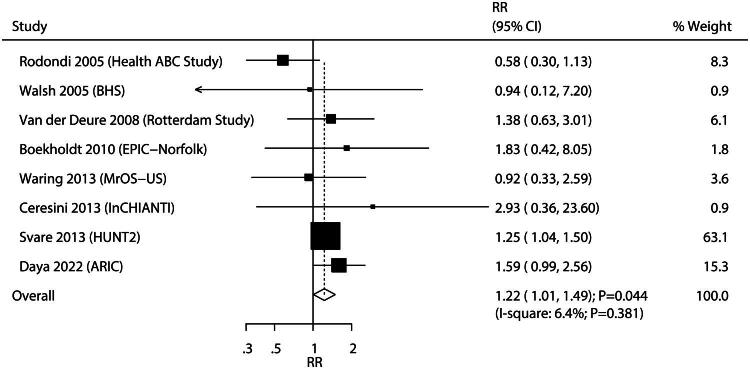
Association of subclinical hypothyroidism with the risk of vertebral fracture.

Eight studies investigated the association between SH and VF risk. SH was associated with an increased risk of VF compared to euthyroidism (RR, 1.22; 95%CI: 1.01–1.49; *p* = 0.044; Figure 3), and no significant heterogeneity was observed among the included studies (*I*^2^ = 6.4%; *p* = 0.381). Sensitivity analysis indicated that the pooled conclusion varied upon sequential removal of individual studies due to a marginal 95% CI (Figure S2). Subgroup analysis found that SH was associated with an elevated risk of VF when pooled studies had a sample size of ≥10,000, median age of <70.0 years, and moderately adjusted levels (Table 1). Moreover, the median age of individuals affected the association between SH and the risk of VF (*p* = 0.047), whereas sample size (*p* = 0.125), proportion of males (*p* = 0.531), follow-up duration (*p* = 0.900), adjusted level (*p* = 0.564), and study quality (*p* = 0.702) did not.

### Publication bias

**Figure 4.**
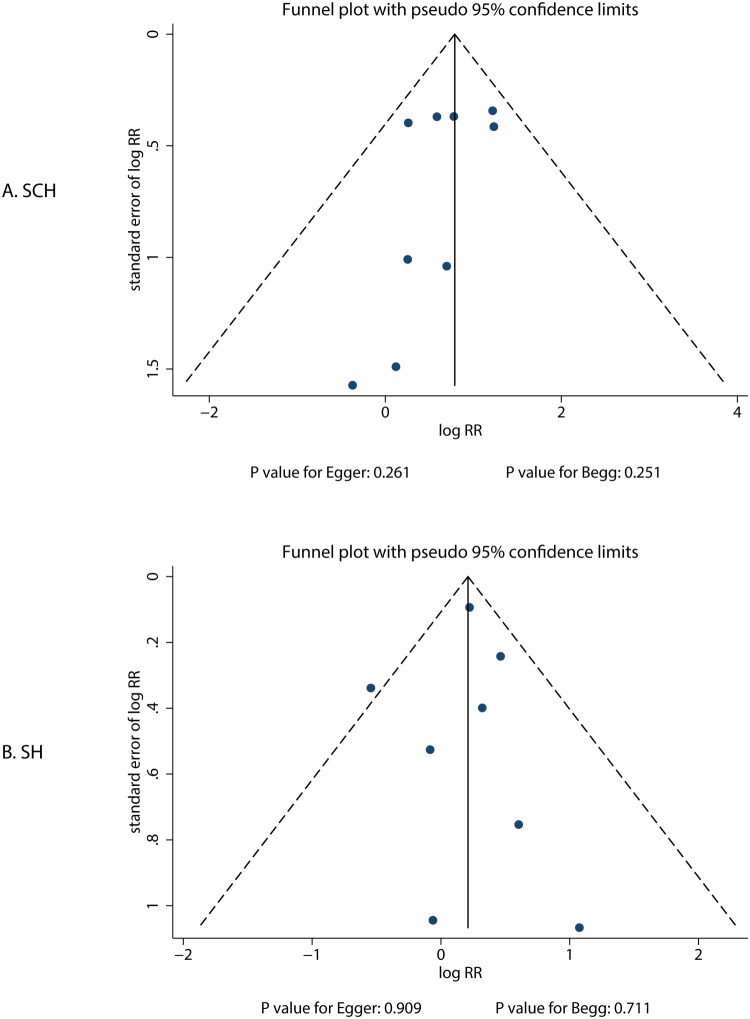
Funnel plots for the associations of subclinical hyperthyroidism (A) and subclinical hypothyroidism (B) with the risk of vertebral fracture.

A review of funnel plots could not rule out potential publication bias (Figure 4). Egger’s and Begg’s test results indicated no significant publication bias for the risk of VFs related to SCH (Egger’s: *p* = 0.261; Begg’s: *p* = 0.251) and SH (Egger’s: *p* = 0.909; Begg’s: *p* = 0.711). Therefore, this study did not employ the trim and fill method to adjust the pooled results.

## Discussion

This systematic review and meta-analysis, which was performed on prospective cohort studies, explored all possible associations between SCH or SH and the risk of VF. A total of 61,219 individuals from 10 prospective cohort studies with a wide range of characteristics were included. This study found that SCH was associated with an increased risk of VF and the pooled conclusions were robust. SH was also associated with an elevated risk of VF; however, the summary results lacked stability due to the marginal 95%CI. Exploratory analyses revealed that the median age of individuals affected the association between SH and VF risk.

Several meta-analyses have reported an association between STD and VF risk [17,19,20]. These previous meta-analyses, however, had limitations in sample size, or the specific populations included, which our current study aims to address by conducting a more comprehensive meta-analysis of prospective cohort studies. Blum et al. found that SCH was not associated with the risk of VF in six prospective cohort studies [17]. This negative finding prompted us to explore this association further with a larger and more diverse sample to determine if the relationship could be different under different circumstances. Yang et al. identified six prospective cohort studies and found that SCH was associated with an increased risk of VF, whereas SH did not affect VF risk [19]. Zhu et al. identified seven prospective cohort studies and found that SCH was associated with an elevated risk of VF, whereas no significant association was observed between SH and VF [20]. Due to the publication of recent studies regarding the association of STD with the risk of VF, the results of the meta-analysis need to be updated. Moreover, further exploratory analysis is needed to investigate whether the risk intensity of STD and VF risk varies among different populations. Our study included a total of 61,219 individuals from 10 prospective cohort studies, which is larger than many previous studies on this topic. This larger sample size allowed us to detect a significant association between SCH and VF risk with greater statistical power, providing more robust evidence compared to previous research that had smaller sample sizes and thus less conclusive results.

The results of this study indicate that SCH is significantly associated with an elevated VF risk. Several reasons could explain this association: (1) the low TSH levels in patients with SCH cause greater bone turnover and bone loss [42,43]; (2) SCH is associated with lower thigh muscle strength and an increased risk of fall-related fractures [44]; and (3) the risk of osteoporosis is elevated in patients with SCH, which is associated with an increased risk of fractures [45,46]. While previous meta-analyses by Yang et al. and Zhu et al. did not find a significant association between SH and VF risk [19,20], our study, with its unique sample characteristics and comprehensive analysis, revealed that SH may indeed increase the risk of VF, especially in certain subgroups such as those with a sample size of ≥10,000 and a median age of <70.0 years. However, the HUNT2 study [39] reported similar results, which accounted for 63.1% of the weight in the meta-analysis. Furthermore, the sensitivity analysis results revealed that the significant association between SH and VF risk was not stable, requiring validation by subsequent large-scale studies. This finding adds a new dimension to the understanding of the relationship between SH and VF and highlights the importance of considering different factors in future research. Nevertheless, the association between SH and increased VF risk may be explained by: (1) The excessive levels of thyroid hormones at the upper limit of the normal range or above can lead to accelerated bone turnover, bone loss, and an increased risk of fractures [47] and (2) metabolic pathways for the role of exogenous and endogenous thyroid hormones differ, and endogenous STD may go undetected for many years because symptoms of STD are often nonspecific or asymptomatic [48].

Subgroup analysis revealed that SCH was associated with an increased risk of VF in most subgroups, except in studies with moderately adjusted levels or moderate quality. A potential reason for this could be the small number of included studies in these subgroups and the fact that the pooled conclusion lacked stability. The significant association between SH and VF was maintained in subgroups with a sample size of ≥10,000, median age of <70.0 years, and studies with moderately adjusted levels. These results could be explained by the following: (1) large sample sizes provided sufficient power to detect a potentially significant association, and the 95% CI was narrow, making it easier to obtain; (2) individuals’ age is significantly associated with osteoporosis, which affects VF risk; and (3) the adjusted level was determined by adjusted factors, which play an important role in the risk of VF.

This study had several limitations. First, the cut-off values for the definitions of SCH and SH were not consistent among the included studies, causing the SCH and SH levels in the comparison population to differ, which could have affected the net effect estimates between STD and the risk of VF. If the cutoff value for SCH is smaller or the cutoff value for SH is larger, the severity of the disease in the STD population will be more severe, and there may be individuals in the control group who meet the criteria for STD according to other standards, which may potentially lead to heterogeneity to some extent. Second, the adjusted factors differed across the included studies, and the effect estimate with a 95%CI for comparisons of SCH or SH and euthyroidism on the risk of VF could be affected by adjusted factors. Third, a previous study found potential sex differences in the association between STD and the risk of fracture [49], whereas a small number of included studies reported an association between STD and the risk of VF according to sex; thus, the sex differences in these associations could not be fully elucidated. Fourth, all of the included studies published in English, and none non-English articles met the inclusion criteria, which could affect the comprehensiveness and representativeness of the findings. Fifth, while publication bias was assessed using funnel plots, Egger’s test, and Begg’s test, it is important to note that these tests have limitations, particularly when the number of studies is small, leading to reduced power to detect bias. Additionally, since the analysis relied on published articles and unpublished data were not accessible, this could have introduced inevitable publication bias. Finally, there are inherent limitations to meta-analyses of published articles, which are based on pooled data, and the detailed analyses are restricted.

## Conclusions

This study found that both SCH and SH are associated with an elevated risk of VFs. The median age of the participants could have affected the association between SH and the risk of VF. The association between SH and the risk of VF requires further verification because the pooled conclusion was not stable. To further clarify the relationship between SH and VF risk, several areas of future research are needed. Larger sample sizes, consistent definitions of SH, and longer follow-up durations are all crucial aspects that should be considered. Future studies addressing these areas can provide more accurate and comprehensive insights into this complex relationship, which will ultimately contribute to better prevention and management of VF in patients with SH.

## Supplementary Material

Figure S1.tif

Table S1.docx

PRISMA Checklist.doc

Figure S2.tif

File S1.docx

## Data Availability

The datasets used and/or analyzed during the current study are available from the corresponding author upon reasonable request.
