# Supplementary figures and images for: Association of subclinical thyroid dysfunction with the risk of vertebral fracture: a meta-analysis of prospective cohort studies
Source: Ann Med. 2025 Sep 11;57(1):2558122. doi: 10.1080/07853890.2025.2558122 (PMC12434856; doi:10.1080/07853890.2025.2558122)

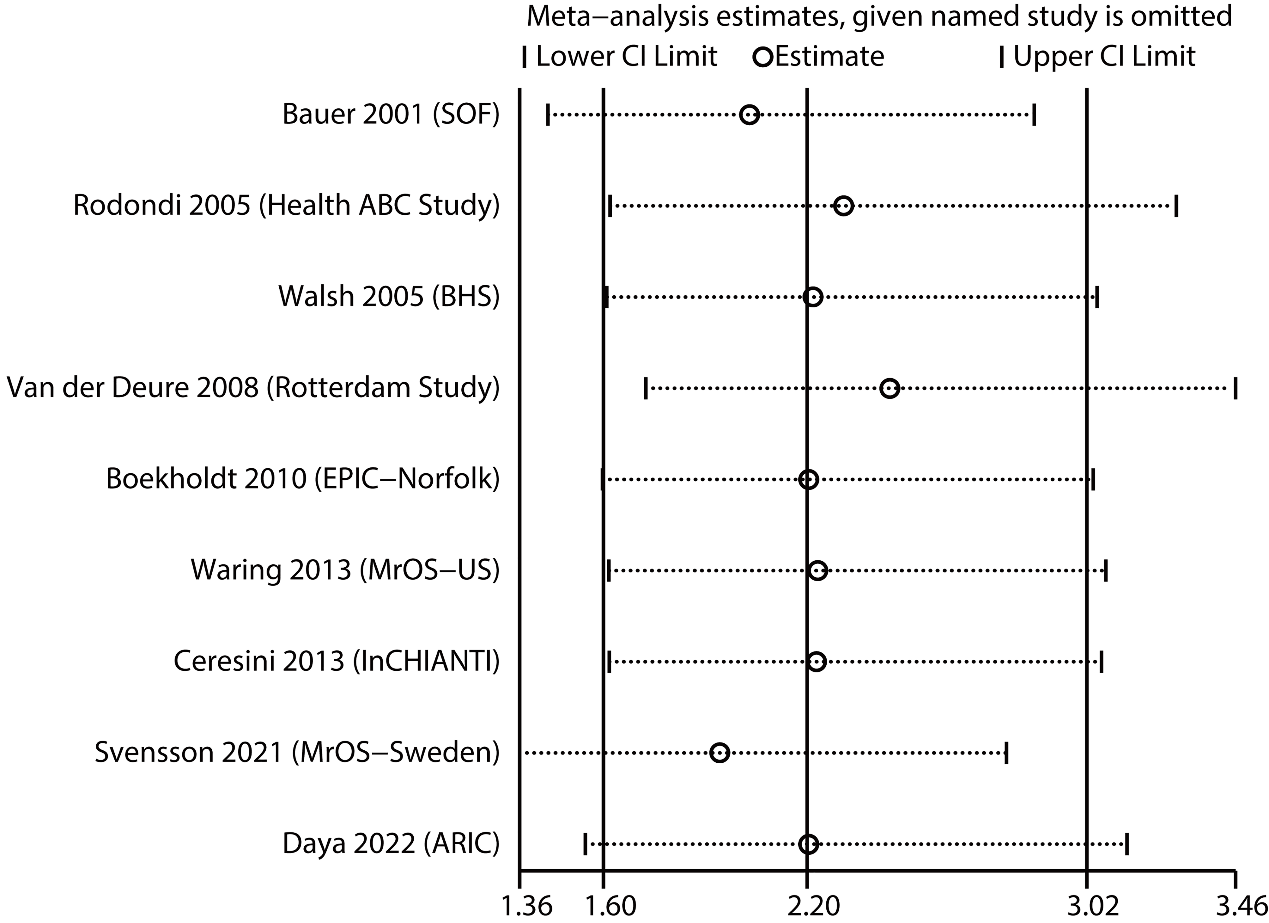

Supplement: Figure S1.tif [file IANN_A_2558122_SM3486.tif]

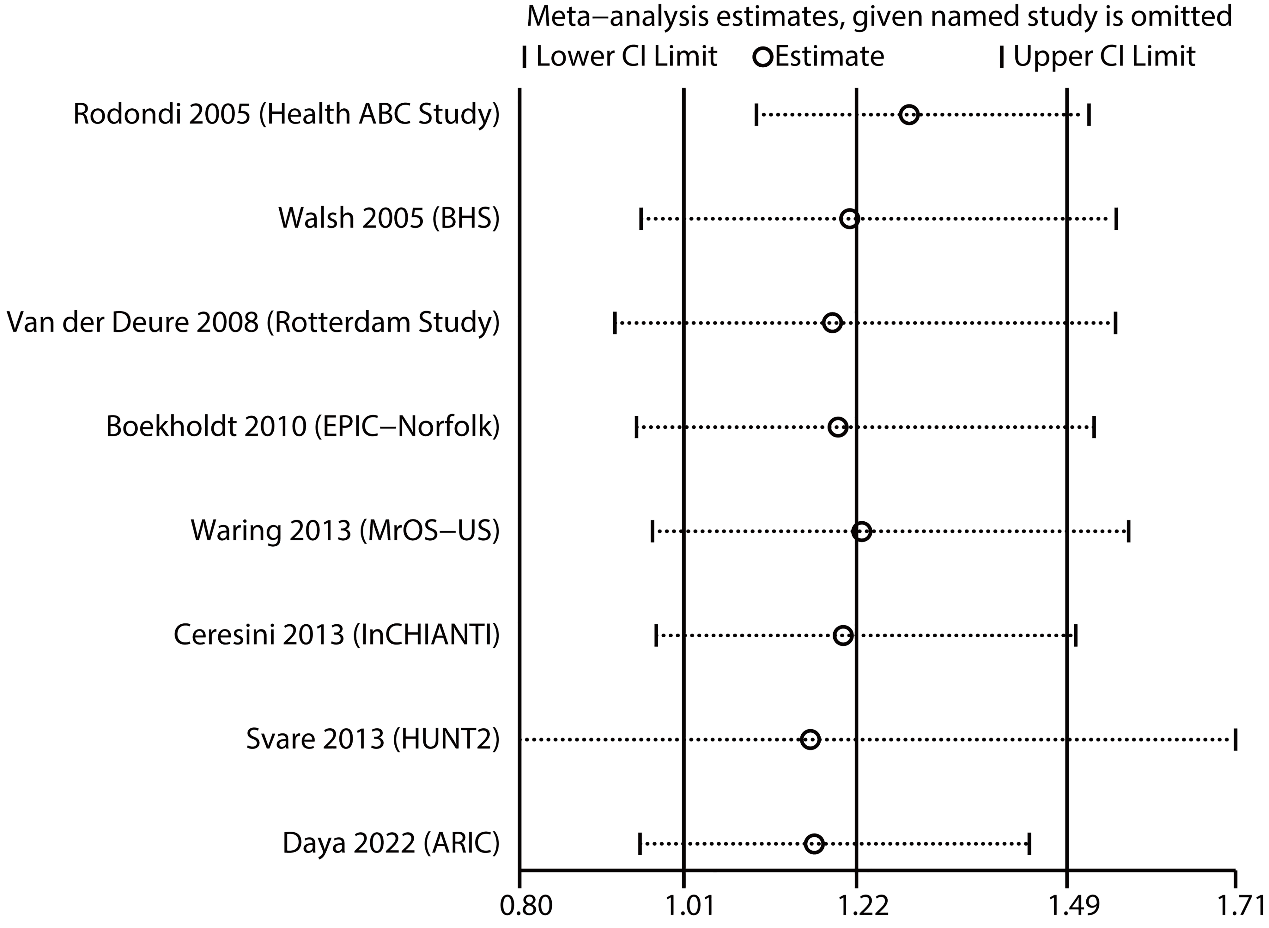

Supplement: Figure S2.tif [file IANN_A_2558122_SM3483.tif]
