# Supplementary material for: Association of subclinical thyroid dysfunction with the risk of vertebral fracture: a meta-analysis of prospective cohort studies
Source: Ann Med. 2025 Sep 11;57(1):2558122. doi: 10.1080/07853890.2025.2558122 (PMC12434856; doi:10.1080/07853890.2025.2558122)
Supplement: Table S1.docx [file IANN_A_2558122_SM3485.docx]

Table S1. The characteristics of included studies and related participants

| Study | Region | Description of study sample | Sample size | Age, median (years) | Male (%) | SCH (mIU/L) | SH (mIU/L) | Thyroid medication | Follow-up duration | Adjusted factors | NOS scale |
| --- | --- | --- | --- | --- | --- | --- | --- | --- | --- | --- | --- |
| Bauer 2001 (SOF) | USA | Four clinical centers | 686 | 72.0 | 0.0 | < 0.5 | - | 10.9 | 3.7 years | Use of thyroid hormone, previous hyperthyroidism, age, self-rated health, and current oral estrogen use | 9 |
| Rodondi 2005 (Health ABC Study) | USA | 2 communities | 2,764 | 74.0 | 49.1 | < 0.45 | > 4.50 | 9.7 | 12.8 years | Age, gender, BMI, smoking status, history of DM, thyroid and thyroid-altering medication, anti-osteoporotic medication | 8 |
| Walsh 2005 (BHS) | Australia | Adults | 2,049 | 51.0 | 50.9 | < 0.45 | > 4.50 | 0.9 | 20.0 years | Age, gender, BMI, smoking status, history of DM, thyroid medication, anti-osteoporotic medication | 7 |
| Van der Deure 2008 (Rotterdam Study) | Netherlands | Adults | 1,838 | 69.0 | 38.7 | < 0.40 | > 4.30 | 2.3 | 9.4 years | Age, gender, BMI, smoking status, history of DM, thyroid medication | 9 |
| Boekholdt 2010 (EPIC-Norfolk) | UK | Adults | 13,066 | 58.0 | 45.6 | < 0.45 | > 4.50 | 3.4 | 12.4 years | Age, gender, BMI, smoking status, history of DM, thyroid and thyroid-altering medication | 8 |
| Waring 2013 (MrOS-US) | USA | 6 clinical centers | 1,513 | 73.0 | 100.0 | < 0.55 | > 4.78 | 7.6 | 8.6 years | Age, clinic site, race, BMI, PA score, alcohol intake, smoking status, corticosteroid use, and thyroid hormone use | 9 |
| Ceresini 2013 (InCHIANTI) | Italy | 2 small towns | 1,186 | 71.0 | 44.0 | < 0.45 | > 4.50 | 2.4 | 9.1 years | Age, gender, BMI, smoking status, history of DM, thyroid and thyroid-altering medication | 7 |
| Svare 2013 (HUNT2) | Norway | Adults | 25,205 | 58.2 | 34.1 | < 0.50 | > 3.50 | 4.7 | 12.5 years | Age, gender, BMI, smoking status, history of DM, thyroid or thyroid-altering medication | 8 |
| Svensson 2021 (MrOS-Sweden) | Sweden | Adults | 1,856 | 75.4 | 100.0 | < 0.45 | - | 1.7 | 8.9 years | Age, MrOS site, levothyroxine treatment, BMI, appendicular lean mass, grip strength, walking speed, smoking status, and total hip sBMD | 9 |
| Daya 2022 (ARIC) | USA | 4 communities | 10,956 | 56.7 | 45.7 | < 0.56 | > 5.10 | - | 21.0 years | Age, gender, race by center, DM, high-density lipoprotein, antihypertensive treatment, heart rate, BMI, smoking status, alcohol intake, PA, menopause, and vitamin D level | 9 |

*BMI: body mass index; DM: diabetes mellitus; PA: physical activity; SH: subclinical hypothyroidism; SCH: subclinical hyperthyroidism
