# Supplementary material for: Association of subclinical thyroid dysfunction with the risk of vertebral fracture: a meta-analysis of prospective cohort studies
Source: Ann Med. 2025 Sep 11;57(1):2558122. doi: 10.1080/07853890.2025.2558122 (PMC12434856; doi:10.1080/07853890.2025.2558122)
Supplement: File S1.docx [file IANN_A_2558122_SM3482.docx]

File S1. Search strategy in PubMed:

("thyroid gland"[MeSH Terms] OR ("thyroid"[All Fields] AND "gland"[All Fields]) OR "thyroid gland"[All Fields] OR "thyroid"[All Fields] OR "thyroid usp"[MeSH Terms] OR ("thyroid"[All Fields] AND "usp"[All Fields]) OR "thyroid usp"[All Fields] OR "thyroids"[All Fields] OR "thyroid s"[All Fields] OR "thyroidal"[All Fields] OR "thyroideal"[All Fields] OR "thyroidism"[All Fields] OR "thyroiditis"[MeSH Terms] OR "thyroiditis"[All Fields] OR "thyroiditides"[All Fields] OR ("thyrotropin"[MeSH Terms] OR "thyrotropin"[All Fields] OR "thyrotropine"[All Fields] OR "thyrotropins"[All Fields]) OR ("hyperthyroidal"[All Fields] OR "hyperthyroidic"[All Fields] OR "hyperthyroidism"[MeSH Terms] OR "hyperthyroidism"[All Fields] OR "hyperthyroid"[All Fields] OR "hyperthyroids"[All Fields] OR "hyperthyroidisms"[All Fields]) OR ("hypothyroidal"[All Fields] OR "hypothyroidic"[All Fields] OR "hypothyroidism"[MeSH Terms] OR "hypothyroidism"[All Fields] OR "hypothyroid"[All Fields] OR "hypothyroidisms"[All Fields] OR "hypothyroids"[All Fields])) AND ("fractur"[All Fields] OR "fractural"[All Fields] OR "fracture s"[All Fields] OR "fractures, bone"[MeSH Terms] OR ("fractures"[All Fields] AND "bone"[All Fields]) OR "bone fractures"[All Fields] OR "fracture"[All Fields] OR "fractured"[All Fields] OR "fractures"[All Fields] OR "fracturing"[All Fields]) AND ("subclinic"[All Fields] OR "subclinical"[All Fields] OR "subclinically"[All Fields] OR "subclinicals"[All Fields])
